# 2K09 and thereafter : the coming era of integrative bioinformatics, systems biology and intelligent computing for functional genomics and personalized medicine research

**DOI:** 10.1186/1471-2164-11-S3-I1

**Published:** 2010-12-01

**Authors:** Jack Y Yang, Andrzej Niemierko, Ruzena Bajcsy, Dong Xu, Brian D Athey, Aidong Zhang, Okan K Ersoy, Guo-zheng Li, Mark Borodovsky, Joe C Zhang, Hamid R Arabnia, Youping Deng, A Keith Dunker, Yunlong Liu, Arif Ghafoor

**Affiliations:** 1 Department of Radiation Oncology, Massachusetts General Hospital Cancer Center and Harvard Medical School, Boston, Massachusetts 02114 U.S.A.; 2 School of Electrical and Computer Engineering, Purdue University, West Lafayette, Indiana 47907 USA; 3 Center for Computational Biology and Bioinformatics, Indiana University School of Medicine, Indiana University Purdue University Indianapolis, Indianapolis, Indiana 46202 USA; 4 Center for Information Technology Research in the Interest of Society, University of California at Berkeley, Berkeley, California, 94720, USA; 5 Department of Computer Science, University of Missouri, Columbia, Missouri 65211-2060 USA; 6 National Center for Integrated Biomedical Informatics (NCIBI) and Center for Computational Medicine and Biology, University of Michigan, Ann Arbor, Michigan 48109 USA; 7 Department of Computer Science and Engineering, University at Buffalo, The State University of New York, Buffalo, New York 14260-2000 USA; 8 Department of Control Science & Engineering, Tongji University, Shanghai 201804 China; 9 Center for Bioinformatics and Computational Genomics, Georgia Institute of Technology, Atlanta Georgia 30332-0535 USA; 10 Department of Computer Science, University of Georgia, Athens, Georgia 30602-7404 USA; 11 The University of Southern Mississippi, Hattisburg, MS 39402, USA; 12 International Society of Intelligent Biological Medicine and SpecPro Inc, 3909 Halls Ferry Road, Vicksburg, MS 39180 USA

## Abstract

Significant interest exists in establishing synergistic research in bioinformatics, systems biology and intelligent computing. Supported by the United States National Science Foundation (NSF), International Society of Intelligent Biological Medicine (http://www.ISIBM.org), International Journal of Computational Biology and Drug Design (IJCBDD) and International Journal of Functional Informatics and Personalized Medicine, the ISIBM International Joint Conferences on Bioinformatics, Systems Biology and Intelligent Computing (ISIBM IJCBS 2009) attracted more than 300 papers and 400 researchers and medical doctors world-wide. It was the only inter/multidisciplinary conference aimed to promote synergistic research and education in bioinformatics, systems biology and intelligent computing. The conference committee was very grateful for the valuable advice and suggestions from honorary chairs, steering committee members and scientific leaders including Dr. Michael S. Waterman (USC, Member of United States National Academy of Sciences), Dr. Chih-Ming Ho (UCLA, Member of United States National Academy of Engineering and Academician of Academia Sinica), Dr. Wing H. Wong (Stanford, Member of United States National Academy of Sciences), Dr. Ruzena Bajcsy (UC Berkeley, Member of United States National Academy of Engineering and Member of United States Institute of Medicine of the National Academies), Dr. Mary Qu Yang (United States National Institutes of Health and Oak Ridge, DOE), Dr. Andrzej Niemierko (Harvard), Dr. A. Keith Dunker (Indiana), Dr. Brian D. Athey (Michigan), Dr. Weida Tong (FDA, United States Department of Health and Human Services), Dr. Cathy H. Wu (Georgetown), Dr. Dong Xu (Missouri), Drs. Arif Ghafoor and Okan K Ersoy (Purdue), Dr. Mark Borodovsky (Georgia Tech, President of ISIBM), Dr. Hamid R. Arabnia (UGA, Vice-President of ISIBM), and other scientific leaders. The committee presented the 2009 ISIBM Outstanding Achievement Awards to Dr. Joydeep Ghosh (UT Austin), Dr. Aidong Zhang (Buffalo) and Dr. Zhi-Hua Zhou (Nanjing) for their significant contributions to the field of intelligent biological medicine.

## Introduction

Bioinformatics, Systems Biology and Intelligent Computing have been extensively used in the advanced research in biomedical, agricultural, environmental, pharmaceutical and medical sciences as well as public health, drug design, genomics and so on. Research and development in these areas are impacting the advances of science and technology. Supported by the U.S. National Science Foundation (NSF), International Society of Intelligent Biological Medicine (ISIBM), International Journal of Computational Biology and Drug Design (IJCBDD) and International Journal of Functional Informatics and Personalized Medicine (IJFIPM), the *ISIBMInternational Joint Conference on Bioinformatics, Systems Biology and Intelligent Computing* (*ISIBM IJCBS 2009*) (http://www.isibm.org/IJCBS) was designed to promote synergistic research and education in bioinformatics, systems biology and intelligent computing. The ISIBM IJCBS 2009 has provided an integrated platform for the cross fertilization of ideas, and to help shape knowledge and scientific achievements by bridging the independent and complementary disciplines into an interactive and attractive forum. ISIBM IJCBS assembles a spectrum of affiliated research workshops, keynote and tutorial lectures and special interest research sessions into a coordinated research meeting held at Tongji University in Shanghai, China on 3–6 August, 2009. The ISIBM IJCBS 2009 was a showcase for integrative research and provided a platform of continuing education with presentations of a number of keynote sessions, workshops, special interest research sessions and tutorial sessions. The conference was also designed for students to learn advanced knowledge in bioinformatics and systems biology for their continued successes in conducting research. Among 300 papers received by the ISIBM IJCBS, a significant portion of the papers were first-authored by students. Each paper was reviewed and ranked by at least three experts in the program and scientific review committee which consists more than one hundred professors in USA, China and worldwide. Over one hundred scholars in the ISIBM IJCBS Program Committee have dedicated themselves to this great academic event in evaluating and selecting papers based on high publication standard. After a rigorous peer review process, 8 papers [1-8] were selected to the *BMC Systems Biology* and 16 papers were selected to the *BMC Genomics*[9-24] from 300+ submitted papers. About 120 papers were selected to the ISIBM IJCBS proceedings published by the IEEE Computer Society.

## Scientific Themes

The *BMC Genomics* and *BMC Systems Biology* supplements for the ISIBM IJCBS consist of 24 research papers, amongst which 8 papers are in the *BMC Systems Biology*[1-8] and 16 papers [9-24] are in *BMC Genomics* research. Those papers include the following scopes.

### 1. Systems biology modeling in molecular biology

Wang et. al. presented dynamic network to model cellular interactions of different cell types [5] (http://www.biomedcentral.com/1752-0509/4/S1/S5). Chowdhary et. al. [4] presented a comprehensive computational analysis on genome wide promoter studies of human histone gene (http://www.biomedcentral.com/1752-0509/4/S1/S4). Wang et. al. [3] presented a novel modelling of protein - nucleic acid interactions on systems biology studies (http://www.biomedcentral.com/1752-0509/4/S1/S3), Chitankar et. al. [7] presented an algorithm for inferring gene regulatory networks from time series DNA microarray data (http://www.biomedcentral.com/1752-0509/4/S1/S7). To fulfil a model based drug development, Wang et. al. [8] proposed a new statistical approach for model based drug development (http://www.biomedcentral.com/1752-0509/4/S1/S8). Jiang et. al. [2] presented a network-based approach that can infer potential microRNAs with disease relevancy (http://www.biomedcentral.com/1752-0509/4/S1/S2). Wang et. al. [10] utilized machine learning to predict the siRNA potencies (http://www.biomedcentral.com/1471-2164/11/S3/S2). Meng et. al. [19] studied inference of the context specific structure and temporal dynamics of gene regulatory network (http://www.biomedcentral.com/1471-2164/11/S3/S11)

### 2. Systematic modelling and prediction of protein structure and function

Xue et. al. [1] presented a comprehensive studies of disordered proteins in archaea, (http://www.biomedcentral.com/1752-0509/4/S1/S1), Zhang et. al. [6] presented a hybrid genetic and tabu search algorithm to predict 3D structure of proteins. (http://www.biomedcentral.com/1752-0509/4/S1/S6). Huang [22] et. al. developed an algorithm for protein studies (http://www.biomedcentral.com/1471-2164/11/S3/S14). Wang et. al. [18] provided advances in clustering methods in proteins. (http://www.biomedcentral.com/1471-2164/11/S3/S10). Lo et. al. [15] presented a databases for proteins (http://www.biomedcentral.com/1471-2164/11/S3/S7).

### 3. Computational cancer studies and modeling

Land et. al. [23] developed new statistical learning approach to improve CT prediction of treatment in patients with metastatic colorectal carcinoma (http://www.biomedcentral.com/1471-2164/11/S3/S15). Dong et. al. [24] investigated genes and miRNAs in glioblastoma (http://www.biomedcentral.com/1471-2164/11/S3/S16). Sun et. al. [13] provided a comparative study of cancer proteins in human protein-protein interaction network (http://www.biomedcentral.com/1471-2164/11/S3/S5).

### 4. Bioinformatis and computational molecular biology studies

Xu et. al. [9] studied pathogens (http://www.biomedcentral.com/1471-2164/11/S3/S1). This paper is part of the plenary banquet keynote lecture of Dr. Dong Xu at the ISIBM IJCBS 2009. Ghosh et. al. [11] studied signal transduction systems (http://www.biomedcentral.com/1471-2164/11/S3/S3). Chowbia et. al. [12] studied rat liver pathways with DNT exposures. (http://www.biomedcentral.com/1471-2164/11/S3/S4). Tang et. al. [17] studied adipose separation of small animals (http://www.biomedcentral.com/1471-2164/11/S3/S9). Zhang et. al. [16] presented a peak detection algorithm for LC/MS (http://www.biomedcentral.com/1471-2164/11/S3/S8).

## Promoting emerging cutting-edge research fields

Bioinformatics, Systems Biology and intelligent computing have been effectively used in the cutting edge translational biomedical research. The resonance and synergy of these fields are enormous, and are having profound impact on the advances of science and medicine. In cooperation with the International Society of Intelligent Biological Medicine http://www.isibm.org, International Journal of Computational Biology and Drug Design (IJCBDD), International Journal of Functional Informatics and Personalized Medicine (IJFIPM), the ISIBM IJCBS 2009 was designed dynamically to promote synergistic research and education. To this end, the ISBM IJCBS program committee especially appreciates the following keynote lecturers:

1) Dr. Dong Xu

James C. Dowell Distinguished Professor, Director of Digital Biology Laboratory and Chair of Computer Science Department, University of Missouri, USA.

*Keynote Lecture Title*: New Frontiers in Translational Research with Next-Generation Sequencing and Proteomics Analyses.

Recent advances in next-generation sequencing and proteomics further empowered translational research in medicine and agriculture. Based on new technologies, systematic approaches for personalized medicine, bioenergy, and transgenic crops are being developed. Dr. Xu showed that these developments have major social and economical impact. New technologies also pushed frontiers towards large-scale studies of more complex systems, such as non-coding RNA, post-translational modifications, and DNA methylations. These studies are typically data rich and require extensive bioinformatics developments. Dr. Xu was the plenary banquet keynote lecturer, he presented a number of examples showing the roles of computer science and bioinformatics in the related developments.

2) Dr. Joydeep Ghosh

Schlumberger Centennial Chair Professor of University of Texas at Austin, USA.

*Keynote Lecture Title*: Locating a Few Useful Clusters in Large Biological Datasets: A Tale of Two Viewpoints.

A key application of clustering data obtained from sources such as microarrays, protein mass spectroscopy and phylogenetic profiles, is the detection of functionally related genes. Typically, only a small number of functionally related genes form meaningful groups, while the rest need to be ignored. Additional complications arise when there are several irrelevant experimental conditions, when the useful clusters occur at different resolutions/scales, and when genes participate in multiple biological processes, leading to multiple cluster memberships. Thus the corresponding data mining problem is to detect a small number of cohesive, possibly overlapping clusters in the data while ignoring irrelevant data portions. Dr. Ghosh discussed two broad approaches to this problem: (a) a generative approach where one determines and fits a suitable probabilistic model to the data, and (b) a non-parametric approach inspired by Wishart’s remarkable but obscure mode analysis work from 1968. The pros and cons of the two approaches have been illustrated by Dr. Ghosh using results from gene expression data analysis. The 2009 ISIBM outstanding achievement award was presented by the committee to Dr. Ghosh right after his plenary keynote lecture.

3) Dr. Aidong Zhang

Professor and Chair of Department of Computer Science and Engineering, University at Buffalo, The State University of New York, USA.

*Keynote Lecture Title*: Computational Analysis of Biological Networks.

The development of computational techniques for the effective analysis of biological datasets is a crucial step in the medical application of bioinformatics. This unique merging of computer science and biomedical expertise is expected to provide the synergy needed to advance biomedical research to the next level. When analyzing biological data, we face many new computational challenges. Algorithms that are specifically designed for biological data are required so that Dr. Zhang can take advantage of their unique features and address the unique problems they raised. In her keynote lecture, Dr. Zhang discussed new computational research issues and approaches to analysis of biological networks, especially protein interaction networks.

The 2009 ISIBM outstanding achievement award was presented to Dr. Zhang by the committee right after her plenary keynote lecture.

4) Dr. Susan M. Bridges

Co-Director of the Institute for Digital Biology and Hearin Distinguished Professor,

Mississippi State University, USA.

*Keynote Lecture Title*: Algorithms for Structural Annotation of Genomes.

Novel approaches for structural annotation of genomes are required. Dr. Bridges extracted the full value from the avalanche of genome sequence generated from new sequencing technologies. New approaches for annotating genomes using proteomics data and for defragmenting dispersed repeats on genomes were described by Dr. Bridges in her plenary keynote lecture.

5) Dr. Zhi-Hua Zhou

Cheung Kong Professor of Nanjing University, China.

*Keynote Lecture Title*: MIML: A New Machine Learning Framework with Application to Drosophila Gene Expression Pattern Annotation.

A new machine learning framework, Multi-Instance Multi-Label Learning (MIML), was proposed recently by Dr. Zhou. In this framework, an object is represented by multiple feature vectors, and is allowed to be associated with multiple class labels simultaneously. His seminal framework was particularly helpful for learning with complicated objects, and has been found well useful in applications such as image categorization and text categorization. The Berkeley Drosophila Genome Project (BDGP) has generated a large amount of gene expression patterns during Drosophila embryogenesis. Automating the annotation process is very desired. However, there is a big challenge; that is, the anatomical and developmental ontology terms are body-part related and present in local regions of images, while in the BDGP, they are attached collectively to groups of images and it is unknown which term is assigned to which region of which image in the group. Dr. Zhou's MIML provided a promising way to address the challenge. His plenary keynote talk gave an introduction to his MIML as well as the application to Drosophila gene expression pattern annotation. The 2009 ISIBM outstanding achievement award was presented to Dr. Zhou by the committee right after his plenary keynote lecture.

6) Dr. Xiaohua Tony Hu

College of Information Science and Technology, Drexel University, USA.

*Keynote Lecture Title*: Mining, Modelling and Evaluation of the Biomolecular Network.

Biomolecular networks dynamically respond to stimuli and implement cellular function, and understanding these dynamic changes is the key challenge for cell biologists. As biomolecular networks grow in size and complexity, the model of a biomolecular network must become more rigorous to keep track of all the components and their interactions, and in general this presents the need for computer simulation to manipulate and understand the biomolecular network model. In his keynote talk, Dr. Hu presented a novel method to mining, modeling and evaluating a regulatory system that executing cellular functions, which can be represented as a biomolecular network. His method consists of two steps. First, a novel scale-free network clustering approach is applied to such biomolecular network to obtain various sub-networks. Second, computational models are generated for the sub-networks and simulated to predict their behavior in the cellular context. He discussed and evaluated some of the advanced computational modeling approaches; in particular, state-space modeling, probabilistic Boolean network modeling, and fuzzy logic modeling. The modeling and simulation results represented hypotheses that were tested against high-throughput biological datasets (microarrays and/or genetic screens) under normal and perturbation conditions. Experimental results on time-series gene expression data for the human cell cycle indicated that Dr. Hu's approach was promising for the sub-network mining and simulation from large biomolecular networks.

7) Dr. Yi Pan

Chair and Professor of Computer Science, Professor of Computer Information Systems, Georgia State University, USA.

*Keynote Lecture Title*: Protein Structure Prediction and its Understanding Based on Machine Learning Methods.

Understanding protein structures is vital to determining the function of a protein and its interaction with DNA, RNA and enzymes. The information about its conformation can provide essential information for drug design and protein engineering. While there are over a million known protein sequences, only a limited number of protein structures were experimentally determined. Hence, prediction of protein structures from protein sequences using computer programs is an important step to unveil proteins’ three dimensional conformation and functions. As a result, prediction of protein structures has profound theoretical and practical influence over biological study. The explanation of how a decision is made during prediction is also important for improving protein structure prediction and guiding the experimental design. In his keynote talk, Dr. Pan showed how to use machine learning methods to improve the accuracy of protein structure prediction and to interpret prediction results. He reported the frontier research on using neural networks, Support Vector Machines combined with Decision Tree and Association Rule for protein structure prediction, rule extraction and prediction interpretation. Evaluation and comparisons of various prediction and rule extraction systems were presented and future research direction in the area were also presented by Dr. Pan.

8) Dr. Patrick Shen-Pei Wang

Professor of Northeastern University and MIT, USA.

*Keynote Lecture Title*: Intelligent Pattern Recognition and Applications to Similarity Measurement.

In his keynote talk, Dr Wang pointed out that time is money, saving time is saving money. Every penny saved is one penny earned as Dr. Wang stressed. His keynote discussed two major concerns that he can consider saving time: communication by post mails, and, transportation on roads, by intelligent pattern recognition, in terms of hierarchical structures and ambiguities. Dr. Wang along with Dr. Xu are the only 2 plenary banquet speakers. His keynote lecture have drawn a lot of interest among large audiences.

9) Dr. Sun Kim

Chair of Bioinformatics Program and Director of Center for Bioinformatics Research, Indiana University School of Informatics, USA.

*Keynote Lecture Title*: A Data-driven, Systems Biology Approach for Exploring the Role of Epigenetics in Drug-resistant Human Cancer.

Dr. Kim's keynote talk summarized a project to investigate the role of epigenetics in the development of the drug resistant phenotype in human cancer, a major research emphasis of the Indiana University and Ohio State University Integrated Cancer Biology. His keynote was a showcase of the translational genomics research at the OSU -IU alliance led by Dr. Tim Hui-ming Huang, a leading cancer biologist and PI of the NIH grants for their collaborative multi-university research projects.

Those keynote lectures showed that in order to understand the mechanisms of live organisms, information must be integrated at multiple levels. ISIBM IJCBS was designed for integrating information from bioinformatics, systems biology and intelligent computing points of views. The synergy of integrative research on both bioinformatics and systems biology using intelligent computing was very beneficial. The ISIBM IJCBS successfully assembled a spectrum of affiliated research workshops, keynote and tutorial lectures and special interest research sessions into a coordinated research meeting held at Tongji University in Shanghai, China on 3–6 August, 2009. The Program and Scientific Committee consists of more than a hundred professors and scholars world-wide and was organized as follows:

Honorary General Chairs

Dr. Ruzena Bajcsy, Member of United States National Academy of Sciences, Institute of Medicine, Member of United States National Academy of Engineering, University Professor of the University of California at Berkeley, USA

Dr. Joydeep Ghosh, Fellow of IEEE, Chair Professor of the University of Texas at Austin, USA

Dr. Michael S Waterman, Member of United States National Academy of Sciences, Fellow of American Academy of Art and Sciences and Academician of French Académie des Sciences. University Professor of the University of Southern California at Los Angles, USA

General Chair

Dr. Mark Borodovsky, President of ISIBM and Regents Professor of Georgia Institute of Technology, USA

General Co-Chair

Dr. Changjun Jiang, Dean of College of Engineering and Information Technology and University Professor of Tongji University, China

Steering Committee Members

1) Dr. Hamid R. Arabnia

Founding chair of WORLDCOMP Congress, Editor-in-Chief of The Journal of Supercomputing (Springer) and recipient of William F. Rockwell, Jr. Medal for promotion of multi-disciplinary research (Rockwell medal is International Technology Institute's highest honor). He is on the editorial and advisory boards of 25 other journals and science magazines.

2) Dr. Ruzena Bajcsy

Member of United States National Academy of Sciences, Institute of Medicine, Member of United States National Academy of Engineering, the 2003-2005 United States President’s Information Technology Advisory Committee (PITAC), 2001 ACM Allen Newell Award, Member of National Institute of Standards and Technology, Member of National Research Council Army Research Technical Assessment Board, Member of Computer Research Association for Women, Member of the Review Board of Stanford University Computer Science, Fellow of ACM and Fellow of IEEE, Distinguished Professor and Director of CITRIS, University of California at Berkeley, U.S.A.

3) Dr. Mark Borodovsky

President of International Society of Intelligent Biological Medicine, Founder of GeneMark and Pioneer of Bioinformatics Research, Founder of Georgia Tech Bioinformatics Ph.D. Programs, Distinguished Regents' Professor and Director Center for the Bioinformatics and Computational Genomics of the Georgia Institute of Technology and Emory University School of Medicine, both in Atlanta, Georgia, USA

4) Dr. Arif Ghafoor

Fellow of IEEE and Professor of School of Electrical and Computer Engineering of Purdue University. Dr. Ghafoor is a leading researcher in multimedia systems, databases, distributed computing systems, broadband multimedia networking and biomedical engineering. Areas of interest include communications, networking, signal & image processing, computer engineering and biomedical engineering.

5) Dr. Joydeep Ghosh

Distinguished Schlumberger Centennial Chair Professor of Engineering, Chair of IEEE DMTC and Fellow of IEEE, 1992 IEEE Darlington Award, Director of IDEAL (Intelligent Data Exploration and Analysis Lab), University of Texas at Austin, U.S.A.

6) Dr. Tony Xiaohua Hu

IEEE Computer Society Bioinformatics and Biomedicine Steering Committee Chair and the IEEE Computational Intelligence Society Granular Computing Technical Committee Chair, the founding Editor-in-Chief of the International Journal of Data Mining and Bioinformatics, an associate editor/editorial board member of four international journals (KAIS, IJDWM, IJSOI and JCIB). Dr. Hu has received a few prestigious awards including the 2005 National Science Foundation (NSF) Career award.

7) Dr. Yi Pan

Editor-in-Chief of the International Journal of Bioinformatics Research and Applications, series editor of Wiley Book Series on Bioinformatics, Associate Editor of 6 IEEE Transactions, winner of many awards, keynote speaker for over 10 conferences, and Chair of Department of Computer Science at Georgia State University, USA

8) Dr. Michael S. Waterman

Member of United States National Academy of Sciences, Fellow of American Academy of Art and Sciences and Academician of French Académie des Sciences. Co Editor-in-Chief of the Journal of Computational Biology, Editor-in-Chief of Lecture Notes in Bioinformatics, Lecture Notes in Computer Science (LNCS, Springer). Distinguished University Professor of University of Southern California, Los Angles, California, USA

9) Dr. Cathy H. Wu

Dr. Wu has conducted bioinformatics research since 1990 and developed several protein classification systems and databases. She has managed large software and database projects, led the bioinformatics effort of the Protein Information Resource (PIR) since 1999, and becoming the PIR Director in 2001. Her research interests include protein family classification and functional annotation, biological data integration, and literature mining.

10) Dr. Mary Qu Yang

Editor-in-Chief, International Journal of Computational Biology and Drug Design. Oak Ridge Institute for Science and Education, Oak Ridge National Lab. U.S. Department of Energy, USA, and National Human Genome Research Institute, National Institutes of Health (NIH) , U.S. Department of Health and Human Services, USA.

11) Dr. Aidong Zhang

Fellow of IEEE and Professor and Chair of Department of Computer Science and Engineering at the State University of New York at Buffalo and the director of the Buffalo Center for Biomedical Computing (BCBC). She is an author of more than 200 research publications and has served on many editorial boards of prestigious journals. Dr. Zhang is a recipient of the National Science Foundation CAREER Award and SUNY (State University of New York) Chancellor’s Research Recognition Award.

Program Chair

Dr. Joe Zhang,

Director of School of Computing, The University of Southern Mississippi, USA

Program Co-Chairs

Dr. Andreas Dress,

Professor and Director, Chinese Academy of Science, CAS-MPG Partner Institute for Computational Biology, Shanghai, China; Professor of University of Bielefeld and Max Planck Institute, Germany and Princeton Institute for Advanced Studies, Princeton, USA;

Dr. Guo-Zheng Li,

Tongji University, China

Publication Chairs

Prof. Heng Huang, University of Texas, Arlington, USA

Prof. Sumanth Yenduri , University of Southern Mississippi, USA

Prof. Zhongming Zhao, Vanderbilt University, USA

Workshop Principal Chairs and Coordinators

Prof. Abdullah N. Arslan, University of Vermont, USA

Dr. Ping Gong, Environmental Laboratory, U.S. Army Engineer Research and Development Center, and SpecPro Inc.

Professor Liangjiang Wang, Clemson University, USA.

Workshop on Bioinformatics Algorithms

Chair: Dr. Abdullah N. Arslan, University of Vermont, USA

Workshop on Bioinformatics in Radiation Oncology and Robotic Interventions in Cancer Diagnosis and Therapy

Chair: Dr. Yan Yu, Professor and Director of Medical Physics, Thomas Jefferson University, USA

Workshop on Machine Learning and Human Diseases

Chairs: Dr. Liangjiang Wang, Clemson University, USA

Dr. Susan M. Bridges, University Professor of the Mississippi State University, USA

Workshop on Bioinformatics and Systems Biology in Traditional Chinese Medicine Research.

Chairs: Dr. Dan Xi, NIH, U.S. Department of Health and Human Services, USA

Dr. Su-Shing Chen, University of Florida, USA.

Workshop on Methods and Applications of Systems Biology

Chairs: Dr. Yidong Chen, University of Texas,

Dr. Yufei Huang, University of Texas

Dr. Ji Zhang, Shanghai Jiao Tong University.

Special Panel on future directions of bioinformatics and systems biology

Chair: Professor Aidong Zhang, State University of New York at Buffalo, USA

Panelists: Professor Susan M. Bridges, Mississippi State University, USA.

Professor Sun Kim, Indiana University, USA.

Professor Yi Pan, Georgia State University, USA.

Professor Bo Yuan, Ohio State University, USA and Shanghai Jiao Tong University, China.

Special Session Principal Chairs and Coordinators

Prof. Jacqueline Signorini, Dean of the Faculty of Sciences, University Paris 8, France

Prof. Tianrui Li, Southwest Jiaotong University, China

Special Session on Evolutionary Systems Biology

Chairs: Prof. Yufeng Wang, University of Texas at San Antonio, USA.

Prof. Jianying Gu, City University of New York, USA.

Special Session on Biomedical Literature Mining and Bioontology

Chair: Dr. Hongfang Liu, Georgetown University, USA

Special Session on Data Mining in Protein Interaction Networks

Chairs: Dr. Xiaoli Li, Nanyang Technological University, Singapore

Dr. Erliang Zeng, University of Notre Dame, Indiana, USA

Special Session on Computational Epidemiology

Chairs: Prof. Armin R. Mikler, University of North Texas, USA

Dr. Courtney Corley, Pacific Northwest National Lab, DOE, USA

Special Session on Linear and Nonlinear Analysis of the EEG in Alzheimer's Disease

Chair: Dr. Jaeseung Jeong, Korea Advanced Institute of Science and Technology, Korea

Special Session on Genomics and Personalize Medicine

Chair: Dr. Leming Zhou, University of Pittsburgh, USA

Special Session on Gene Networks and Pathway Analysis

Chairs: Prof. Zhongming Zhao, Vanderbilt University, USA.

Prof. Danail Bonchev, Virginia Commonwealth University, USA

Tutorial Chair

Prof. Shuigeng Zhou, Fudan University, China

Tutorial Lecture on Pattern Discovery in Genomics Context

Speaker: Professor Yi-Ping Phoebe Chen, Deakin University, Australia

Invited Lecture on Latent Structure Models and Diagnosis in Traditional Chinese Medicine

Invited Speaker: Professor Nevin L. Zhang, Hong Kong University of Science and Technology, Hong Kong.

Publicity Chairs

Dr. Jiaoxiong Xia, Shanghai Municipal Education Commission, China

Prof. Yilong Yin, Shandong University, China

Dr. Mehdi Pirooznia, Johns Hopkins University, Baltimore, Maryland, USA

Dr. Yongqing Su, Tongji University, Shanghai, China

Registration Chairs

Prof. Youping Deng, University of Southern Mississippi and SpecPro Inc, USA

Prof. Fei Qiao, Tongji University, China

Organizing Chairs

Dr. Weisheng Xu, Tongji University, China

Dr. Youling Yu, Tongji University, China

Dr. Mingyu You, Tongji University, China

Dr. Preetam Ghosh , University of Southern Mississippi, USA

Award Chairs

Prof. Susan Bridges, Mississippi State University, USA

Prof. Hamid R. Arabnia , University of Georgia, USA

Prof. Yong-Sheng Ding , Donghua University, China

Prof. Jie Yang, Shanghai Jiao Tong University, China

Prof. Duoqian Miao, Tongji University, China

Local Chair

Dr. Jing Yao, Tongji University, China

Committee Members

1. Dr. Sadaf ALAM, Oak Ridge National Laboratory, USA

2. Prof. Laurence Rodrigues Do AMARAL, Federal University of Goias, Brazil

3. Dr. Purushotham BANGALORE, University of Alabama at Birmingham, USA

4. Dr. Pierre BEAUSEROY, University of Technology in Troyes, UTT, France

5. Dr. Mahua BHATTACHARYA, Indian Institute of Information Technology & Management, India

6. Dr. Hong CAI, University of Texas at San Antonio, USA

7. Rui CAMACHO, Porto University, Portugal

8. Prof. Zhiwei CAO, Tongji University, China

9. Cornelia CARAGEA, Iowa State University, USA

10. Dr. Doina CARAGEA, Kansas State University, USA

11. Dr. Rui CHANG, University of California, USA

12. Dr. Bernard CHEN , University of Central Arkansas, USA

13. Prof. Jake Y. CHEN, Indiana University Purdue University Indianapolis, USA

14. Dr. Yidong CHEN, University of Texas Health Science Center at San Antonio, USA

15. Dr. Jianlin (Jack) CHENG, University of Missouri, USA

16. Dr. Yanfeng CHENG, Georgia Institute of Technology, USA

17. Dr. Qiang CHENG, Southern Illinois University Carbondale, USA

18. Prof. Sung-Bae CHO, Yonsei University, Korea

19. Dr. Albert C. S. CHUNG , Hong Kong University of Science and Technology, Hong Kong

20. Dr. Mark CLEMENT, Brigham Young University, USA

21. Dr. Kevin DAIMI, University of Detroit Mercy, USA

22. Dr. Phuongan DAM , University of Georgia, USA

23. Prof. Yisheng DING, Donghua University, China

24. Dr. Qiwen DONG, Fudan University, China

25. Dr. Pan DU, Northwestern University, USA

26. Prof. Ye DUAN, University of Missouri - Columbia, USA

27. Dr. Werner DUBITZKY, University of Ulster at Coleraine, UK

28. Prof. Minrui FEI, Shanghai University, China

29. Dr. Elisa FICARRA, Politecnico di Torino, Italy

30. Christopher M. FRENZ, New York City College of Technology (CUNY)

31. Dr. Jean X. GAO , University of Texas at Arlington, USA

32. Prof. Yang GAO, Nanjing University, China

33. Dr. Xin GENG, South East University, China

34. Dr. Preetam GHOSH , University of Southern Mississippi, USA

35. Dr. Kreshna GOPAL, Texas A&M University, USA

36. Prof. Jianying GU , City University of New York, USA

37. Dr. Jun-tao GUO , The University of North Carolina at Charlotte, USA

38. Prof. Maozu GUO, Harbin Institute of Technology, China

39. Dr. Yufei Huang, University of Texas at San Antonio, USA

40. Prof. Wen-Lian HSU, Institute of Information Science, Academia Sinica, Taiwan

41. Dr. Hongwei HUO, Xidian University, China

42. Dr. Jaeseung JEONG, Korea Advanced Institute of Science and Technology, Korea

43. Dr. Liangxiao JIANG, University of Geosciences, China

44. Dr. Rui JIANG, Tsinghua University, China

45. Dr. Zhenran JIANG, East China Normal University, China

46. Prof. Jaewoo KANG , Korea University, Korea

47. Abdellali KELIL, University of Sherbrooke, Canada

48. Dr. Dongsup KIM , Korea Advanced Institute of Science and Technology, Korea

49. Dr. Hyunsoo KIM, Harvard University, USA

50. Dr. Sun KIM, Indiana University, USA

51. Dr. Jiejun KONG, University of California, USA

52. Dr. Wei KONG, Shanghai Maritime University, China

53. Prof. Rui KUANG , University of Minnesota, USA

54. Dr. Zoé LACROIX, Arizona State University, USA

55. Prof. Feipei LAI, National Taiwan University, Taiwan

56. Dr. Chang-Shing LEE , National University of Tainan, Taiwan

57. Dr. Haim LEVKOWITZ, University of Massachusetts at Lowell, USA

58. Dr. Chang-Tsun LI , University of Warwick, UK

59. Dr. Jia LI, Shanghai University, China

60. Dr. Kang LI, Queen's University Belfast, UK

61. Dr. Liao LI, University of Delaware, USA

62. Dr. Ming LI, Nanjing University, China

63. Prof. Shao LI, Tsinghua University, China

64. Prof. Shaozi LI, Shamen University, China

65. Dr. Shen LI, Indiana University, USA

66. Prof. Tianrui LI, Southwest Jiaotong University, China

67. Dr. Xiaoli LI, Nanyang Technological University, Singapore

68. Dr. Xiaolin LI, Nanjing University, China

69. Dr. Ying-Xin LI, Nanjing University, China

70. Dr. Tim. G. LILBURN, American Type Culture Collection, USA

71. Dr. Chun-Yuan LIN , Chang Gung University

72. Dr. Simon LIN, Northwestern University Biomedical Informatics Center, USA

73. Dr. Hongfang LIU, Georgetown University, USA

74. Dr. Jun LIU, Nanjing University of Aeronautics and Astronautics, China

75. Dr. Qingzhong LIU, New Mexico Tech, USA

76. Dr. Tianming LIU, The University of Georgia, USA

77. Prof. Gary LIVINGSTON, University of Massachusetts at Lowell, USA

78. Dr. Dang LONG, New York State Department of Health, USA

79. Prof. Hongtao LU, Shanghai JiaoTong University, China

80. Prof. Wencong LU, Shanghai University, China

81. Prof. Carol LUSHBOUGH, University of South Dakota, USA

82. Dr. Xiaotu MA, Shanghai Jiao Tong University, China

83. Dr. Ian M. MACDONALD, The College of Saint Rose, USA

84. Dr. Anant MADABHUSHI, Rutgers University, The State University of New Jersey, USA

85. Dr. Krzysztof MALCZEWSKI, Poznan University of Technology, Poland

86. Prof. Kezhi MAO, Nanyang Technological University, Singapore

87. Dr. Majid MASSO , George Mason University, USA

88. Dr. Vasilis MEGALOOIKONOMOU, Temple University, USA

89. Prof. Duoqian MIAO, Tongji University, China

90. Prof. Armin R. MIKLER, University of North Texas, USA

91. Dr. Nasir-ud-Din, Institute of Molecular Sciences & Bioinformatics, Pakistan

92. Dr. Jun NI , University of Iowa, USA

93. Dr. Giuseppe NICOSIA, University of Catania, Italy

94. Timothy O'CONNOR, University of Cambridge, UK

95. Prof. Manish PALIWAL, Southern Illinois University at Carbondale, USA

96. Minseo PARK, University of Massachusetts, USA

97. Dr. Vinhthuy PHAN , University Of Memphis, USA

98. Dr. Helen PIONTKIVSKA, Kent State University, USA

99. Dr. Aleksandar POLEKSIC, University of Northern Iowa, USA

100. Monika RAY, Washington University in St. Louis, USA.

101. Dr. Tong RUAN, East China University of Science and Technology, China

102. Dr. Ann RUNDELL, Purdue University, USA

103. Meena K. SAKHARKAR, Nanyang Technological University, Singapore

104. Prof. Jacqueline SIGNORINI, University Paris 8, France

105. Dr. Xiaofeng SONG, Nanjing University of Aeronautics and Astronautics, China

106. Dr. Vojislav STOJKOVIC, Morgan State University, USA

107. Dr. Jonathan SUN, University of Southern Mississippi, USA

108. Prof. Xiao SUN, Southeast University, China

109. Dr. Jay URBAIN, Illinois Institute of Technology, USA

110. Prof. Athanasios VASILAKOS, University of Peloponnese, Greece

111. Dr. Thanos VASILAKOS, University of Western Macedonia, Greece

112. Dr. Miguel A. VEGARODRIGUEZ, University of Extremadura, Spain

113. Dr. Jason T. L. WANG, New Jersey Institute of Technology, USA

114. Dr. Liangjiang WANG, Clemson University, USA

115. Dr. Min WANG, Hohai University, China

116. Dr. Nan Wang, University of Southern Mississippi, USA

117. Dr. Ruizhi WANG, Tongji University, China

118. Dr. Yufeng WANG, University of Texas at San Antonio, USA

119. Dr. Yuhang WANG, Southern Methodist University, USA

120. Dr. Yu-Ping WANG, University of Missouri-Kansas City, USA

121. Dr. Zhe WANG, East China University of Science and Technology, China

122. Dr. Qishi WU , University of Memphis, USA

123. Dr. Jiaoxiong XIA, Shanghai Municipal Education Commission, China

124. Dr. Min XU, University of Southern California, USA

125. Prof. Weisheng XU, Tongji University, China

126. Dr. Jianhua XUAN , Virginia Tech, USA

127. Dr. Kaiguo YAN, Thomas Jefferson University, USA

128. Prof. Jie YANG, Shanghai JiaoTong University, China

129. Prof. Laurence T. YANG , St Francis Xavier University, Canada

130, Prof. Yilong YIN, Shandong University, China

131. Dr. Jingkai YU, Wayne State University, USA

132. Dr. Yan YU, Thomas Jefferson University, USA

133. Dr. Erliang ZENG, University of Miami, USA

134. Prof. Aidong ZHANG, State University of New York at Buffalo, USA

135. Dr. Daoqiang ZHANG, Nanjing University of Aeronautics and Astronautics, China

136. Dr. Ji Zhang, Shanghai Jiao Tong University School of Medicine, China

137. Dr. Jun ZHANG, University of Kentucky, USA

138. Dr. Min-Ling ZHANG, Hohai University, China

139. Dr. Weixiong ZHANG, Washington University, USA

140. Dr. Yanqing ZHANG, Georgia State University, USA

141. Dr. Ying ZHAO, Tsinghua University, China

142. Dr. Huiru(Jane) ZHENG, University of Ulster, UK

143. Dr. W. Jim ZHENG, Medical University of South Carolina, USA

144. Prof. Yang ZHONG, Fudan University, China

145. Dr. Jie ZHOU, Northern Illinois University, USA

146. Dr. Leming ZHOU, University of Pittsburgh, USA

147. Prof. Shuigeng ZHOU, Fudan University, China

148. Dr. Xiaobo ZHOU, Cornell University, USA

149. Prof. Zhi-Hua ZHOU, Nanjing University, China

150. Dr. Hill ZHU, Florida Atlantic University, USA

151. Prof. Qingxin ZHU, University of Electronic Science and Technology, China,

152. Dr. Shanfeng ZHU, Fudan University

The International Society of Intelligent Biological Medicine awards 1-3 scholars for the Outstanding Achievement Awards each year based on following criteria: 1) Substantiated outstanding contributions to the field of intelligent biological medicine; 2) Research of the highest level; 3) Significant scientific impact; 4) Scientific leadership. At the banquet of the conference, the scientific committee presented the 2009 ISIBM Outstanding Achievement Awards to Dr. Joydeep Ghosh (UT Austin), Dr. Aidong Zhang (Buffalo) and Dr. Zhi-Hua Zhou (Nanjing) for their significant contributions to the field of intelligent biological medicine after their plenary keynote presentations. Since 2007 ISIBM has awarded 11 scholars for the Outstanding Achievement Awards: The 2007 awards were presented to Dr. Ruzena Bajcsy (UC Berkley), Dr. Mary Qu Yang (NIH) and Dr. Chin-Mingh Ho (UCLA) at the IEEE International Conference on Bioinformatics and Bioengineering at Harvard Medical School in Boston, Massachusetts USA, and the 2008 awards were presented to Dr. Brian D. Athey (Michigan) and Dr Jack Yang (Harvard) at the World Congress on Computer Science, Computer Engineering and Applied Computing in Las Vegas, Nevada USA. Furthermore, by the time of this paper is published in November 2010, the scientific committee has also presented the 2010 ISIBM Outstanding Achievement Awards to Dr. Arif Ghafoor (Purdue), Dr. Mark Borodovsky (Georgia Tech) and Dr. Dong Xu (University of Missouri) for their significant contributions to the field of intelligent biological medicine. The non-profit academic society welcomes the nominations for the 2011 awards. Please send your nominations to President of ISIBM T. K. Li Professor A. Keith Dunker, Vice-President Professor Hamid R. Arabnia and Secretary-General Professor Yunlong Liu. 1-3 winners will be announced by the president of the society after the rigorous evolution and selection process by the committee.

## Conclusions

As upcoming emerging fields, bioinformatics, systems biology and intelligent computing integrate scientific and engineering knowledge to solve significant biomedical problems. It is important to train the next generation bioinformatics and systems biology experts with up-to-date technologies and advancements in the fields. ISIBM IJCBS 2009 provided an opportunity to promote inter/multidisciplinary scientific research and education for students, faculty, scientists, engineers and medical doctors, and help them better prepared for new initiatives and cutting-edge researches. Updated cutting-edge technologies and breaking through ideas were presented in the keynote and tutorial lectures, in the open discussion sessions, and in special interest and scientific exchange sessions to inspire innovations, novel ideas and scientific discoveries. The *BMC Genomics* and the *BMC Systems Biology* issues were products of part of the achievement of the ISIBM International Joint Conferences on Bioinformatics, Systems Biology and Intelligent Computing in promoting emerging cutting-edge research fields with most updated research trends, novel technologies, breakthrough ideas and scientific discoveries. We hope that attendees' experiences with the professional society were, are and will be always pleasant, memorable and productive. The non-profit academic society strives to promote science and education and warmly welcomes suggestions world-wide.

## Conflict of Interest

The authors declared no conflict of interest.
